# Imaging of Neuronal Activity in Awake Mice by Measurements of Flavoprotein Autofluorescence Corrected for Cerebral Blood Flow

**DOI:** 10.3389/fnins.2017.00723

**Published:** 2018-01-04

**Authors:** Manami Takahashi, Takuya Urushihata, Hiroyuki Takuwa, Kazumi Sakata, Yuhei Takado, Eiji Shimizu, Tetsuya Suhara, Makoto Higuchi, Hiroshi Ito

**Affiliations:** ^1^Department of Functional Brain Imaging Research, National Institutes for Quantum and Radiological Science and Technology, Chiba, Japan; ^2^Division of Thermo-Biosystem Relations, United Graduate School of Agricultural Science, Iwate University, Morioka, Japan; ^3^Department of Cognitive Behavioral Physiology, Graduate School of Medicine, Cognitive Behavioral Therapy Center Research Center for Child Mental Development, Chiba University, Chiba, Japan; ^4^Advanced Clinical Research Center, Fukushima Global Medical Science Center, Fukushima Medical University, Fukushima, Japan

**Keywords:** flavoprotein autofluorescence imaging, intrinsic optical signal imaging, cerebral blood flow, oxygen metabolism, image correction method for green fluorescent imaging

## Abstract

Green fluorescence imaging (e.g., flavoprotein autofluorescence imaging, FAI) can be used to measure neuronal activity and oxygen metabolism in living brains without expressing fluorescence proteins. It is useful for understanding the mechanism of various brain functions and their abnormalities in age-related brain diseases. However, hemoglobin in cerebral blood vessels absorbs green fluorescence, hampering accurate assessments of brain function in animal models with cerebral blood vessel dysfunctions and subsequent cerebral blood flow (CBF) alterations. In the present study, we developed a new method to correct FAI signals for hemoglobin-dependent green fluorescence reductions by simultaneous measurements of green fluorescence and intrinsic optical signals. Intrinsic optical imaging enabled evaluations of light absorption and scatters by hemoglobin, which could then be applied to corrections of green fluorescence intensities. Using this method, enhanced flavoprotein autofluorescence by sensory stimuli was successfully detected in the brains of awake mice, despite increases of CBF, and hemoglobin interference. Moreover, flavoprotein autofluorescence could be properly quantified in a resting state and during sensory stimulation by a CO_2_ inhalation challenge, which modified vascular responses without overtly affecting neuronal activities. The flavoprotein autofluorescence signal data obtained here were in good agreement with the previous findings from a condition with drug-induced blockade of cerebral vasodilation, justifying the current assaying methodology. Application of this technology to studies on animal models of brain diseases with possible changes of CBF, including age-related neurological disorders, would provide better understanding of the mechanisms of neurovascular coupling in pathological circumstances.

## Introduction

To better understand the mechanisms of brain function during resting and stimulation states, green fluorescence imaging (e.g., GCaMP calcium indicator and flavoprotein autofluorescence imaging, FAI) has been used in many biological and medical studies (Nakai et al., 2001; Shibuki et al., 2003). In particular, using *in vivo* animal models, green fluorescence imaging allows us to estimate acute and longitudinal neural and astroglial activity. Intrinsic optical signal imaging (IOSI) and voltage-sensitive dye imaging (VSDI) are also used to evaluate brain function. However, IOSI measurement mainly indicates cerebral blood volume (CBV) (Martin et al., 2006; Ma et al., 2013), and cannot be used to estimate neural activation directly. VSDI is not sensitive enough to accurately observe activated brain regions in small animals, and it is not suitable for longitudinal measurement because an invasive dye-injection process is required during each experiment.

Flavoprotein autofluorescence imaging (FAI) has recently been utilized to capture the changes in mitochondrial oxidative metabolism in the brain (Husson et al., 2007; L'Heureux et al., 2009; Sirotin and Das, 2010). Since neuronal activity is closely related to aerobic energy metabolism and oxygen consumption (Shibuki, 1989; Malonek and Grinvald, 1996), examining oxygen metabolism in the brain is useful for understanding the mechanism of brain functions.

Hemoglobin in cerebral blood vessels absorbs green fluorescence during brain functional imaging. In particular, the cerebral blood flow (CBF) response to neural activity increases the absorption of flavoprotein autofluorescence with a wavelength of 525 ± 25 nm (Vazquez et al., 2012). The possibility exists that the CBF response could greatly affect the signals of green fluorescence obtained from the neuronal and astroglial activation. However, because the onset of an increase in CBF, evoked by neural activation, is generally later than that in FAI, we can obtain the original FAI signal at an earlier time point after neural activation. Thus, the signal change of FAI would be consistent with changes of neuronal activation in the healthy mouse brain (Shibuki et al., 2003). In addition, CBF response-evoked neural activity generally shows both short- and long-term high reproducibility in healthy mice (Takuwa et al., 2011). Therefore, we hypothesized that the interference by CBF due to fluorescence absorption during green fluorescence imaging can be ignored for the estimation of brain function in normal animals.

On the other hand, previous studies have indicated that animal models of stroke and dementia have an attenuated CBF response to neural activation (Iadecola, 2004; Tajima et al., 2014). As a result of the reduction of absorption of fluorescence in cerebrovascular diseases, there is a possibility that the change in signals of green fluorescence during neural activation will be overestimated. Thus, in the case of animal models of brain disease, it may be difficult to accurately estimate brain function using green fluorescence imaging. For this reason, in the present study, we developed a correction method for green fluorescence imaging to remove the effects of light absorption on the signal during neural activation. The basic concept of the compensation method is as follows. IOSI generally represents the reduction rate of reflection light with the light absorption effects of CBF. Therefore, if we simultaneously measure FAI and IOSI in animal brain, the light absorption effects of CBF on FAI signals may be corrected with IOSI signals. Finally, we successfully demonstrated accurate estimation of brain function regardless of the hemodynamics, indicating that this method is useful for estimating brain function in animal models of stroke and dementia.

## Materials and methods

### Animal preparation

A total of 15 male C57BL/6J mice (20–30 g, 7–11 weeks; Japan SLC, Inc., Hamamatsu) were used for simultaneous measurements of FAI and IOSI. These mice were housed with *ad libitum* food and water in their cages at 25°C in a 12-h light/dark cycle. All experiments were performed in accordance with the institutional guidelines on humane care and use of laboratory animals and were approved by the Institutional Committee for Animal Experimentation of National Institutes for Quantum and Radiological Science and Technology.

The animals were anesthetized using a mixture of air, oxygen, and isoflurane (3–5% for induction and 2% for surgery) via a facemask, and a thinned-skull window (5 mm in diameter) was attached over the left somatosensory cortex (including the somatosensory barrel cortex). The previously reported thinned-skull window creation method (Takuwa et al., 2011) was improved and implemented. A midline incision (10 mm) was made to expose the skull over the left somatosensory cortex. The skull (3 by 3 mm centered at 1.8 mm caudal and 2.5 mm lateral from the bregma) was thinned to translucency using a dental drill. The thinned skull was coated with a layer of cyanoacrylate glue and then covered with a cover glass (5 mm in diameter). Dental cement was applied around the edges of the coverslip to stabilize the cover glass to the skull (Shih et al., 2012). A custom metal plate was affixed to the skull through a 7-mm-diameter hole centered over the cranial window. After completion of the surgery, the animals were allowed to recover from anesthesia and housed for at least 1week before initiation of the experiments.

### Experimental protocol

The experimental protocol for measurements using awake mice was reported previously (Takuwa et al., 2011, 2012, 2013a,b). Briefly, the metal plate on the animal's head was screwed to a custom-made stereotactic apparatus. The animal was then placed on a styrofoam ball that was floated using a stream of air. This allowed the animal to exercise freely on the ball while its head was fixed to the apparatus. Under this condition, FAI and IOSI measurements in the somatosensory cortex were performed.

Simultaneous measurements of FAI and IOSI were performed under three separate measurement conditions: (1), sensory stimulation, (2) 5% CO_2_ inhalation, (3) sensory stimulation 10 s after CO_2_ inhalation. To validate our correction method we compared the corrected FAI signals measured under different stimulation and CO_2_ inhalation condition. Although the increase in CBF under hypercapnia greatly reduces the FAI signal, it is expected that the corrected FAI time activity curve will be the same with or without hypercapnia because the level of nerved activity does not change (Matsuura et al., 2000).

### Simultaneous measurement of FAI and IOSI

**Figure 1:**
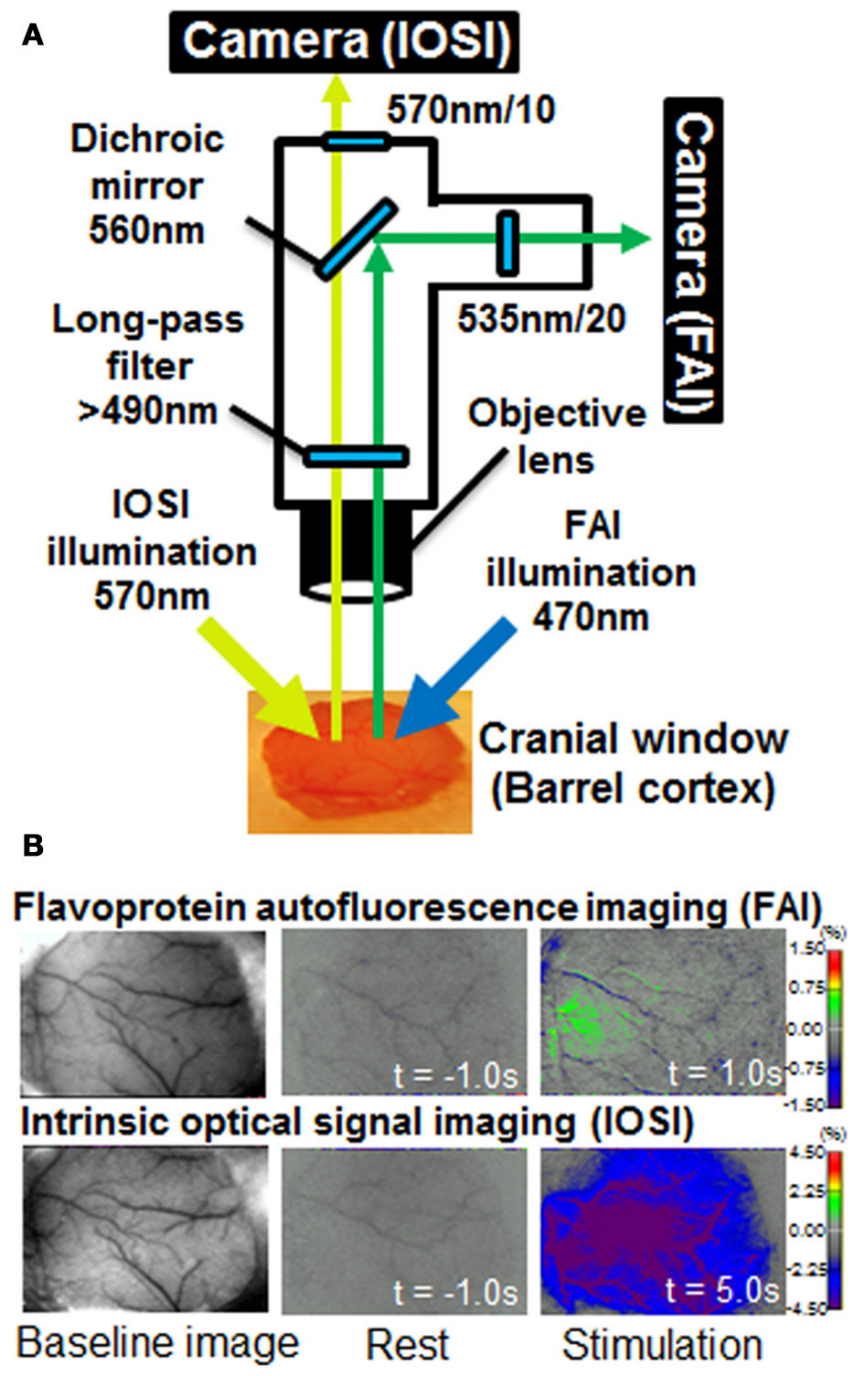
**(A)** Diagram of the experimental apparatus used for simultaneous flavoprotein autofluorescence imaging (FAI) and intrinsic optical signal imaging (IOSI). The exposed cortical surface in the cranial window was simultaneously illuminated with light from two halogen sources passing through two types of band pass filter (wavelength: 470 and 570 nm). For IOSI measurements, reflected light from the cortical surface was reflected to a camera via a 560-nm dichroic mirror. A 570 ± 10 nm filter was placed in front of the camera. For FAI measurements, autofluorescence, filtered by a longpass filter (>490 nm) and reflected by a dichroic mirror (<560 nm), was introduced to a charged couple device (CCD) camera through a band-pass filter (535 nm). FAI and IOSI signals were simultaneously captured using two MiCAM02 CCD cameras. **(B)** Typical images of FAI and IOSI measurements. Each image shows baseline, rest (*t* = −1 s) and stimulation (*t* = 1 or 5 s) from the left. The rest and stimulation images indicate the rate of change relative to the mean value at resting state (10 s).

**Figure 2:**
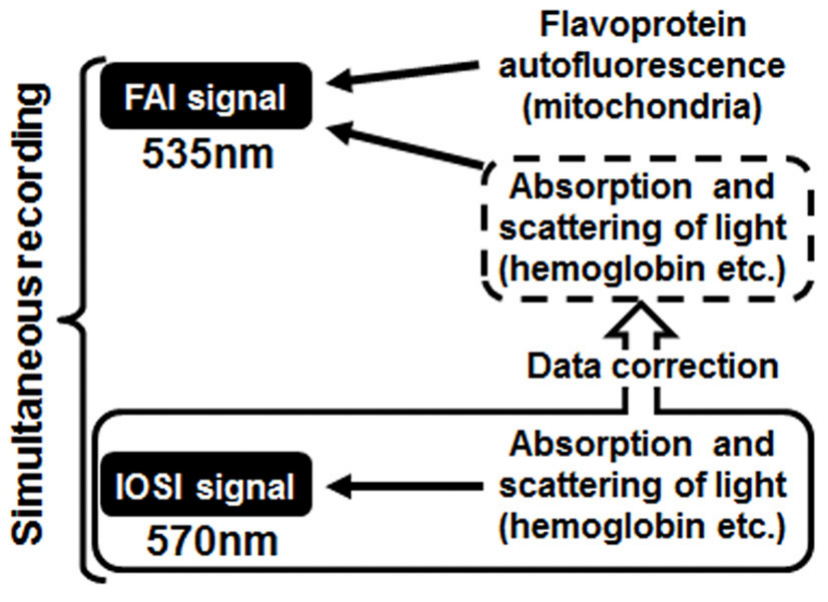
Schematic diagram of the correction method. The absorption and scattering effects of cerebral blood flow contained in the flavoprotein autofluorescence imaging (FAI) signal were removed using a converted value calculated from intrinsic optical signal imaging, which was measured at the same time as FAI.

Takuwa et al. (2014) previously described a custom setup for the simultaneous measurement of FAI and IOSI (Figure 1). FAI and IOSI were simultaneously performed using two CCD cameras (MiCAM02, Brainvision, Tokyo, Japan) (Figure 1A). Temporal resolution and spatial resolution were 10 Hz for 25 s (250 frames/trial) and 192 × 128 pixels (each pixel size was 15 × 15 μm). A total of 25 trials were successively performed with an inter-trial interval of 30 s, and the image was averaged over the trials to improve the signal-to-noise ratio. The exposed cortical surface in the cranial window was simultaneously illuminated with light from two halogen lamps passing through two types of band pass filters (wavelength: 470 and 570 nm). For IOSI measurements, a reduction rate of reflection at 570 nm, which is an isosbestic wavelength of hemoglobin, can allow the measurement of total hemoglobin and is closely correlated to CBV if hematocrit remains constant and proportional to CBF (Martin et al., 2006; Zhao et al., 2009; Ma et al., 2013). In FAI measurements, the autofluorescence emitted from brain cells was collected with an objective lens. Autofluorescence, filtered by a longpass filter (>490 nm) and reflected by a dichroic mirror (<560 nm), was introduced to a MiCAM02 CCD camera through a band-pass filter (535 nm) (Figure 1). Since the flavoprotein fluorescence is at 535 nm, which is also an isosbestic wavelength of hemoglobin, the measured FAI is mainly the sum of autofluorescence and the absorption of light with hemoglobin (Figure 2).

### Compensational method for FAI using IOSI data

**Figure 3:**
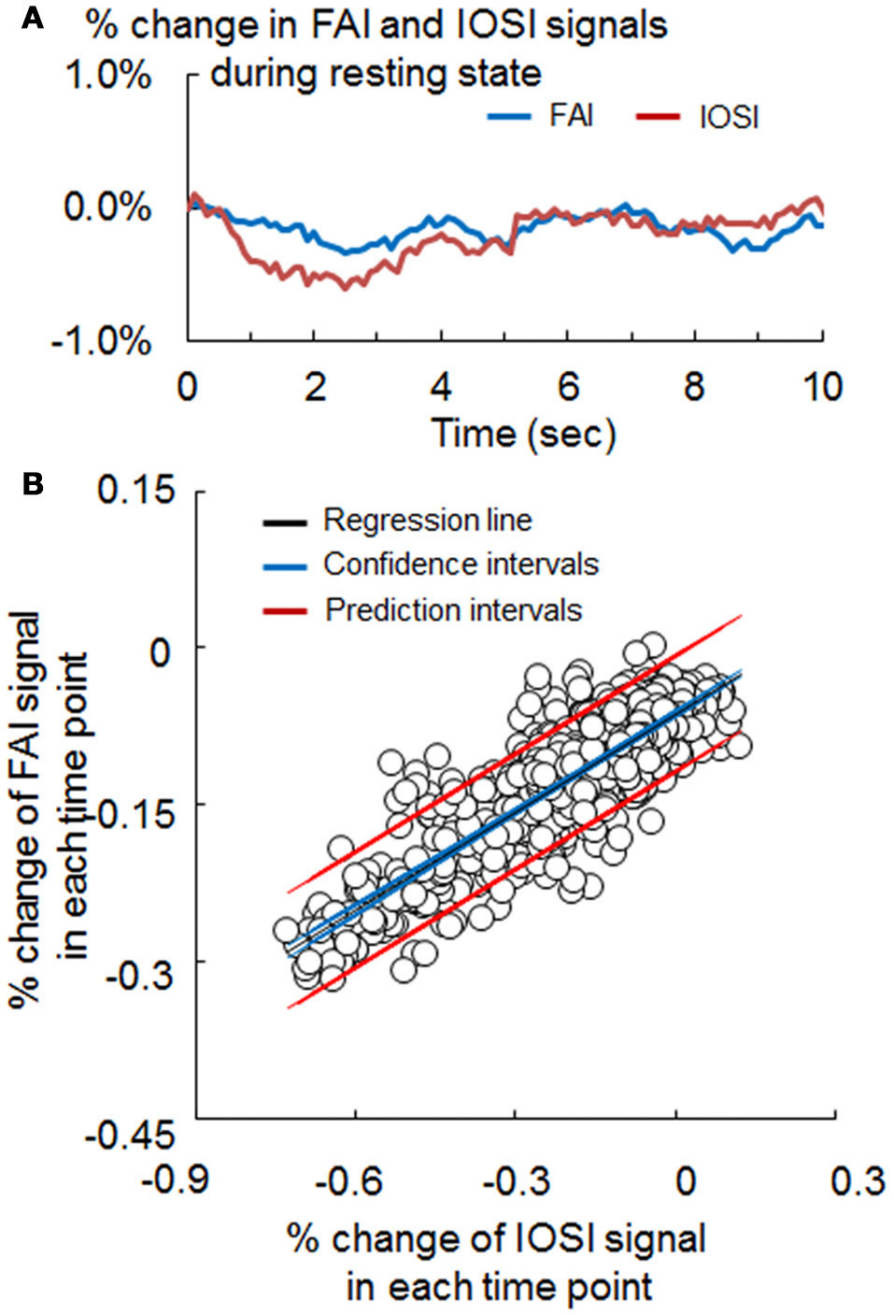
**(A)** Signal change of flavoprotein autofluorescence imaging (FAI; blue) and intrinsic optical signal imaging (IOSI; red) in the resting state, obtained by simultaneous measurement. **(B)** Scatter plot of the FAI and IOSI signals during resting state. The coefficient (α) used for the correction method was obtained from the slope of the approximation straight line (black). Confidence intervals (blue) and prediction intervals (red) are also drawn on the graph.

An uncorrected FAI signal contains not only the flavoprotein autofluorescence but also the absorption and scattering effects with CBF. The purpose of this method was to remove these effects of CBF on FAI and accurately estimate the original flavoprotein autofluorescence caused by neuronal activity (Figure 2). At first, a scatter plot was created from the values of FAI and IOSI signals at each time point during the resting state; we calculated an approximate straight line, confidence interval and prediction interval from a scatter plot using the Curve Fitting Toolbox of MatLab (MathWorks, MA, USA) (Figures 3A,B).

The corrected FAI is expressed as follows:

Corrected FAI = FAI − α × IOSI, where α is the slope of the approximate line equation, and FAI and IOSI data are simultaneously obtained during resting state. We calculated the corrected FAI in all frames (250) and pixels (192 × 128) for each experiment.

### Sensory stimulation and hypercapnia

In sensory stimulation experiments, the hemodynamic response to neuronal activation was induced by sensory stimulation. An air puff was delivered to all of the right whiskers of the mice at a pressure of ~15 psi using a compressed-air bottle. Rectangular pulse stimulation (50-ms pulse width and 100-ms onset-to-onset interval, i.e., 10 Hz frequency) generated with a Master-8 (A.M.P.I., Jerusalem, Israel) was induced for a 2-s duration (Takuwa et al., 2011).

In hypercapnia experiments, the hemodynamic response to hypercapnia was induced by 5% CO_2_ inhalation. A hypercapnic gas mixture of 5% CO_2_, 21% O_2_, and residual N_2_ was inhaled by awake-behaving mice via a facemask (300 ml/min). At all times except during the CO_2_ inhalation, the mice inhaled room air (300 ml/min). CO_2_ gas was given to the mice using the same time schedule as in the sensory stimulation (experiment 1), i.e., 10-s pre-inhalation, 2-s CO_2_ inhalation, and 30-s post-inhalation periods, using the Master-8 (Figure 1). CO_2_ inhalation was repeated 25 times at 60-s intervals after post-stimulus periods, and all trials of the FAI and IOSI signals were averaged offline.

### Data analysis

The magnitudes of increases in the measurements of FAI and IOSI during whisker stimulation were calculated as the mean percentage change from baseline over a 10-s resting state period. A circle with a diameter of 1 mm around the peak of the signal change was chosen as the region of interest (ROI), and the time–response curves of FAI and IOSI were recorded from the mean in the ROI. Statistical analyses were performed with a paired *t*-test and Pearson product-moment correlation coefficient.

## Results

### Simultaneous measurements of FAI and IOSI

Figure 1 shows a schematic diagram of the experimental apparatus for simultaneous FAI and IOSI, and representative data from before (resting condition) and after whisker stimulation. The increase in FAI was observed within 1 s after whisker stimulation. The reduction of a signal in IOSI appeared 5 s after the stimulation, a result of the increasing light absorption associated with the CBF response to neural activation. The activated regions of IOSI (5 s) and FAI (1 s) were observed in almost the same brain area, although the size of the active region in IOSI was larger than that in FAI.

### Comparison between signals of FAI and IOSI during resting state

The waveforms of FAI and IOSI during resting state are shown in Figure 3A. To evaluate the relationship between these parameters, we investigated the correlation between FAI and IOSI during resting state. A linear relationship between the percentage changes in FAI and IOSI during resting state was revealed (Figure 3B). The FAI signal showed a positive correlation with the IOSI signal during resting state (Table 1). Using a scatter plot of FAI and IOSI data, corrected FAI was calculated.

**Table 1:** Relationship between FAI and IOSI signals in each pixel at resting state.

**Animal no**.	**Regression line**	**Correlation coefficient**
Animal 1	*y* = 0.60*x* + 0.36	*r* = 0.82
Animal 2	*y* = 0.45*x* + 0.51	*r* = 0.83
Animal 3	*y* = 0.91*x* + 0.29	*r* = 0.78
Animal 4	*y* = 0.76*x* + 0.35	*r* = 0.74

### Comparison between FAI and corrected FAI during neural activation

**Figure 4:**
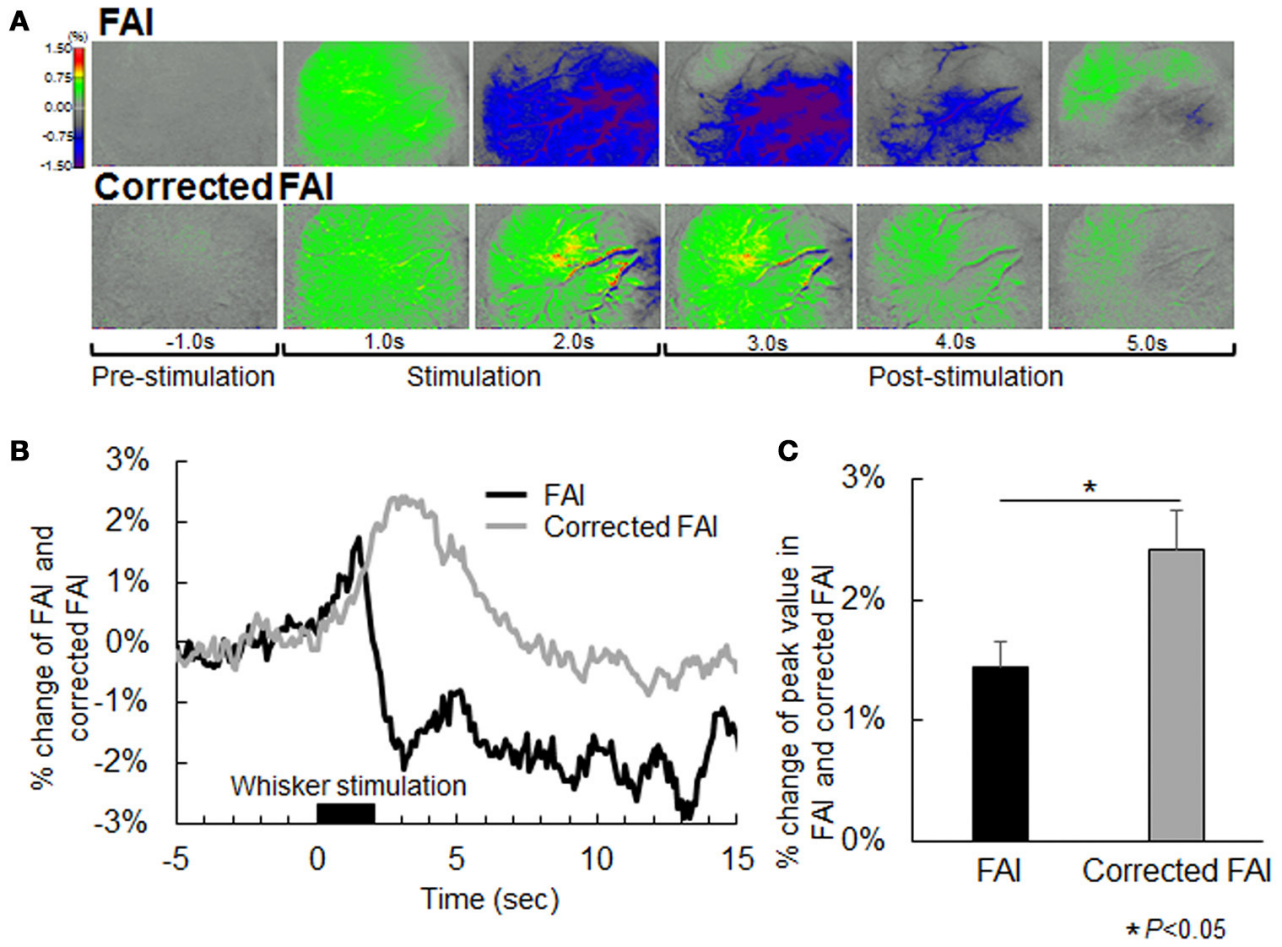
**(A)** Flavoprotein autofluorescence imaging (FAI; top) and corrected FAI images (bottom) from one representative animal. Nerves of each animal were activated by air stimulation to the whiskers. Each image is indicated as pre-stimulation (−1.0 s), stimulation period (1.0 s and 2.0 s), and post-stimulation (3.0, 4.0, and 5.0 s) from the left. The decrease in signal observed by uncorrected FAI was not confirmed by corrected FAI. **(B)** Averaged time-response curves of FAI and corrected FAI from all animals. Black bar indicates duration of whisker stimulation. **(C)** Average of peak value of signal change rate of FAI (black) and corrected FAI (gray) from all animals. Peak value of the corrected FAI signal was significantly higher than that of FAI (*P* < 0.05).

The representative FAI and corrected FAI before (resting state) and after sensory stimulation are shown in Figure 4A. Although the area of increase in FAI appeared 1 s after stimulation, the active area in FAI disappeared, declining from the baseline level 2 s after stimulation (Figure 4A). On the other hand, in the case of corrected FAI, the area of increase in FAI was maintained during sensory stimulation (Figure 4A). The time–response curve of FAI exhibited an initial peak at an earlier time point than that of corrected FAI (Figure 4B). The peak value of FAI was significantly lower than that of corrected FAI (Figure 4C).

### Validation of our correction method (hypercapnia during resting state)

**Figure 5:**
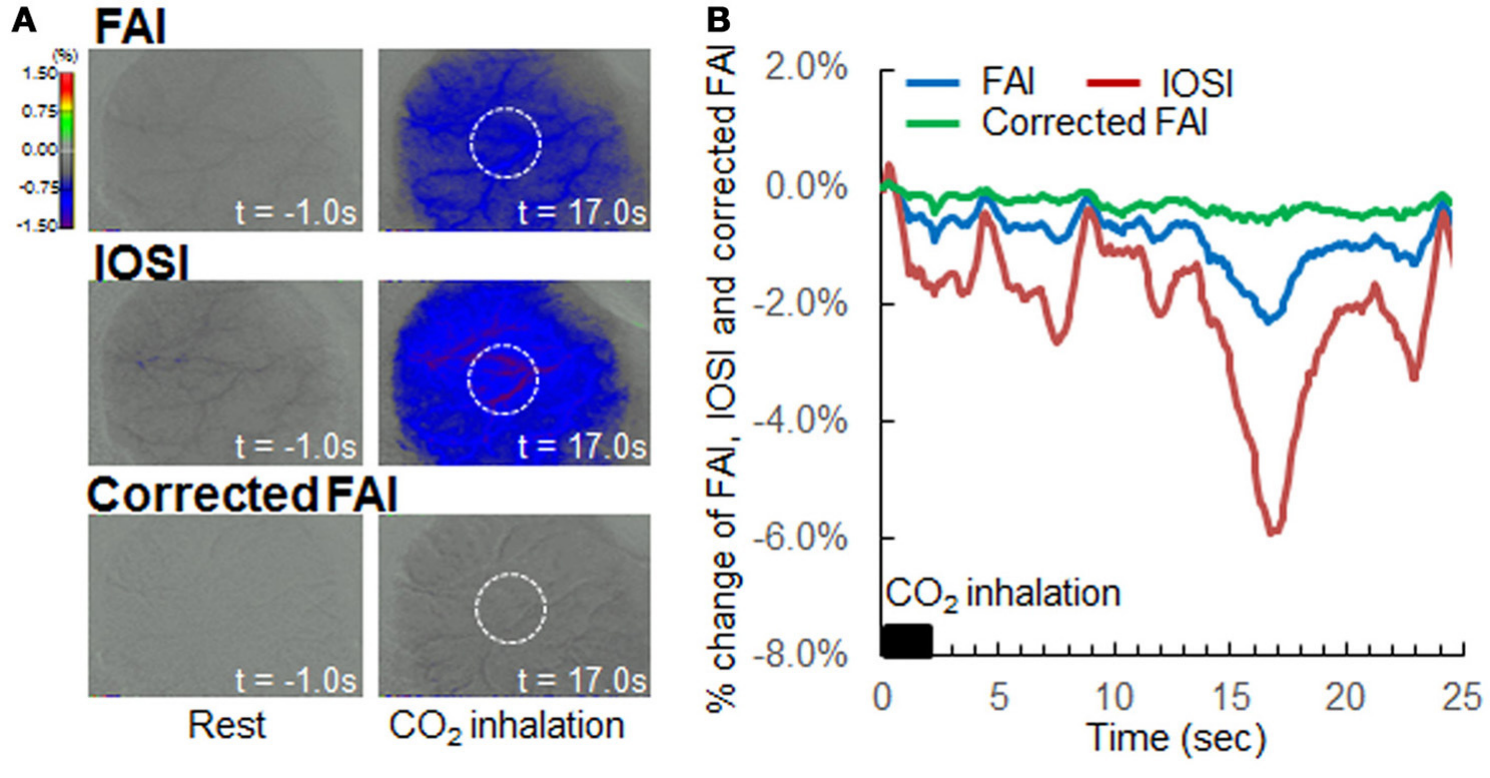
Verification of correction method by hypercapnia during resting state. **(A)** Flavoprotein autofluorescence imaging (FAI; top), intrinsic optical signal imaging (IOSI; middle), corrected FAI (bottom) images from one representative animal. Resting state (left) and hypercapnia state as a result of 5% CO_2_ inhalation (right) are indicated. Circles drawn with white dotted lines indicate regions of interest. **(B)** Averaged time-response curves of FAI (blue), IOSI (red), and corrected FAI (green) signals from all animals. Black bar indicates duration of CO_2_ inhalation. Corrected FAI showed no signal reduction by hypercapnia, and the signal plateaued for the duration of the experiment.

In previous reports, hypercapnia condition by 5% CO_2_ inhalation resulted in an increase in CBF but did not change neural activation (Matsuura et al., 2000). If there are no effects of light absorption with CBF on FAI, the time-response curve of FAI will show a plateau during and after hypercapnia condition. However, light absorption with CBF decreased the FAI signal in this study. Using our correction method, the FAI signal plateaued for the entire measurement period (Figure 5).

### Validation of our correction method (hypercapnia during neural activation)

**Figure 6:**
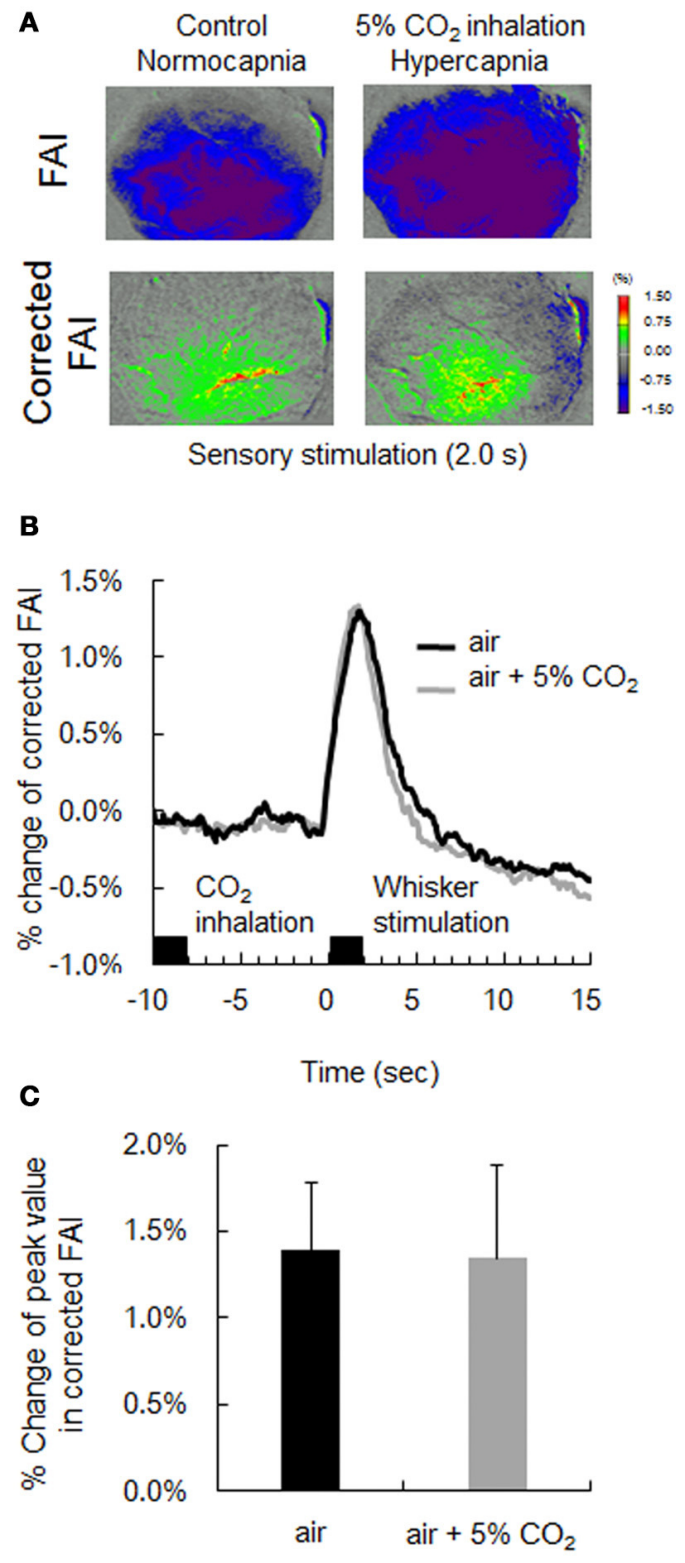
Verification of correction method by hypercapnia during neural activation. **(A)** Flavoprotein autofluorescence imaging (FAI; top) and corrected FAI (bottom) images during sensory stimulation (2.0 s) from one representative animal. Animals were given only sensory stimulation (left) or both sensory stimulation and CO_2_ inhalation (right). **(B)** Averaged time-response curves of corrected FAI signal from all animals. Black bar indicates duration of CO_2_ inhalation and whisker stimulation. **(C)** Average peak value of signal change rate of only air puff (black) and both air puff and 5% CO_2_ inhalation (gray) of all animals. There were no significant differences.

In this experiment, mice were subjected to both 5% CO_2_ inhalation and whisker stimulation to validate our correlation method. As presented in Figure 6A, an increase in CBF with hypercapnia suppressed the increase in FAI during neural activation, and the active area of FAI after stimulation disappeared. On the other hand, using our method, the time response curve of corrected FAI with hypercapnia was consistent with that of corrected FAI without hypercapnia (Figure 6B). There were no significant differences in the change of peak value between the corrected FAI with and without hypercapnia (Figure 6C).

## Discussion

We newly developed a correction method for FAI in an animal study. Our correction method could cancel out the light absorption effects on change in FAI with an increase in CBF during neural activation. To the best of our knowledge, this is the first method to obtained original time activity curve of FAI during neural activity under normal physiological condition in awake mouse brain. Increase in CBF during neural activity is caused by the neurovascular unit in the brain (Attwell et al., 2010). Thus, cerebrovascular dysfunction leads directly to a decrease in light absorption by CBF, resulting in an inaccurate assessment of brain function by green fluorescence imaging. The correction method developed in the present study provides a way of solving the technical problem of the attenuating effects of CBF on green fluorescence imaging during neural activation.

Our results, as presented in Figure 1B, showed that signal changes of FAI and IOSI were observed in almost the same brain region. As a result, the peak amplitude of uncorrected FAI was smaller than that of corrected FAI during neural activation, suggesting that the amount of oxygen metabolism or neural activation is underestimated in uncorrected FAI. This result indicated that, in the case of healthy animals, our correction method is necessary for accurate measurement of brain function.

Comparison of neural function before and after the onset of disease is important for more clearly understanding the mechanism of brain disorders in animal models. For example, we previously showed that the CBF response to sensory stimulation was attenuated 1 week after chronic hypoperfusion caused by unilateral common carotid artery occlusion (CCAO) (Nishino et al., 2016). Our future studies will focus on the measurement of neural activation in animal models of stroke and dementia. However, as a result of a decrease in CBF response after CCAO, uncorrected FAI will overestimate the neural activation after occlusion. We hypothesized that the same technical problem will exist in green fluorescence imaging in several animal models of brain disease. Vazquez et al. (2012) previously demonstrated that inhibition of additional cerebral vasodilation from the baseline level using drug administration (vasodilatory agent sodium nitroprusside) allows us to obtain the original time activity curve of FAI during neural activity. The previous study indicated that increase in FAI signal continuously maintained within sensory stimulation after inhibition of CBF response with drug administration. The time activity curve of FAI obtained with our correction method in this study was in good agreement with the results of drug administration by Vazquez et al. (2012). On the other hand, a condition without drug administration is preferable for accurate measurement of brain function. Our results indicated that, although the increase in CBF under hypercapnia greatly attenuated the signal of FAI during both resting state and sensory stimulation, the time activity curve of corrected FAI was the same with and without hypercapnia. These results indicated that, if animal models of cerebrovascular disease are used, our correction method is essential for the accurate measurement of brain function using green fluorescence imaging.

Although light scattering and absorption mainly contributed to the reduction of FAI, their causes are still unclear. The structure of the brain is too complex to allow a complete identification of all elements causing a change in fluorescence with the current technology. One of the important advantages of our method is that we corrected FAI using the actual measurement value (IOSI) as an index of light scattering and absorption. Therefore, although we could not identify all elements of light scattering and absorption, we could calculate the corrected FAI. In addition, there is no need to consider the abundance ratio of oxygenated and reduced-type hemoglobin in this correction method, because both wavelengths of FAI and IOSI are isosbestic points of hemoglobin. However, spontaneous neuronal activation during the resting state may cause fluctuations in the FAI signal.

It is well known that two-photon imaging using a GCaMP calcium indicator can detect neural activation at a single-cell level. Unfortunately, our correction method is not suitable for high spatial-resolution microscopy because two-photon microscopy separates and detects nerve cells and blood vessels independently. However, the field of view in two-photon imaging is too small to compare the activities of multiple brain regions. To perform wide-field functional *in vivo* imaging, single-photon imaging with a charge-coupled device (CCD) or a complementary metal-oxide semiconductor camera is still being used in animal studies (Vanni and Murphy, 2014). We think that our correction method will be useful for not only FAI, but also wide-field functional *in vivo* imaging using a fluorescence microscope with a CCD camera and a GCaMP indicator.

In conclusion, we developed a new correction method for green fluorescence imaging using the simultaneous measurement system of FAI and IOSI. Our correction method can remove the attenuation effects of CBF on FAI during neural activation. We conclude that the application of our correction method to brain disease-model animals could provide a better understanding of the mechanisms of neurovascular coupling and age-related brain disorders including stroke and dementia.

## Author contributions

HT and HI designed the research; MT, TU, and HT performed the research; MT, TU, KS, and HT analyzed the data; YT, ES, MH, and TS helped with the data interpretation and discussion; MT, TU, HT, MH, and HI wrote the paper.

### Conflict of interest statement

The authors declare that the research was conducted in the absence of any commercial or financial relationships that could be construed as a potential conflict of interest.
